# Non-Pharmacological Treatment Methods of Lennox–Gastaut Syndrome—Review of the Literature

**DOI:** 10.3390/biomedicines13092247

**Published:** 2025-09-12

**Authors:** Piotr Duda, Michał Granat, Stanisław J. Czuczwar, Barbara Miziak

**Affiliations:** 1 Student Scientific Group, Department of Pathophysiology, Medical University of Lublin, ul. Jaczewskiego 8b, 20-090 Lublin, Poland; 2 Department of Pathophysiology, Medical University of Lublin, ul. Jaczewskiego 8b, 20-090 Lublin, Poland; stanislaw.czuczwar@umlub.pl

**Keywords:** drug-refractory epilepsy, epileptic encephalopathy, corpus callosotomy, vagus nerve stimulation, dietary therapy

## Abstract

Lennox–Gastaut syndrome (LGS) is a severe form of childhood-onset epilepsy, often associated with pharmacoresistance. As complete seizure control is usually not achievable with the use of drug therapy, non-pharmacological treatment may be offered to intractable patients. In this review, we are going to present literature reports on various non-pharmacological treatments, including surgical and dietary methods. Surgical interventions, such as resective surgery, corpus callosotomy (CC), or neuromodulation therapies such as vagus nerve stimulation (VNS), deep brain stimulation (DBS), and responsive neurostimulation (RNS), can be offered to pharmacoresistant patients. If the epileptogenic area can be detected, resective surgery is a treatment of choice. On the contrary, if non-invasive and invasive diagnostic methods fail to detect epileptogenic lesions, CC and VNS are considered palliative surgical methods. While both CC and VNS are considered effective in seizure reduction, CC is still more popular than VNS, although VNS seems to be related to better tolerability. Although all neuromodulation therapies require multidirectional optimization, DBS appears to be particularly promising for LGS. The classic ketogenic diet (cKD) is considered an effective and well-tolerated method in LGS treatment. The modified Atkins diet (MAD) and the low glycemic index treatment (LGIT) could be used as valuable alternatives due to their lower restrictiveness and better tolerability. Moreover, combinations of several treatment methods could significantly improve LGS patients’ seizure outcomes.

## 1. Introduction

Lennox–Gastaut syndrome (LGS) is a type of childhood-onset epileptic syndrome associated with severe seizures and intellectual disability [1]. Most cases present before eight years of age, usually between three and five years [2]. Clinical presentation of the syndrome is a triad of drug-resistant seizures (particularly tonic and atypical absence seizures), a characteristic pattern of electroencephalography (EEG), and cognitive impairment [3]. The prevalence of LGS is 1–2% of all patients with epilepsy and 1–10% of childhood epilepsy [3]. The etiology of LGS can be divided into two groups. Most patients (65–75%) have an identifiable cause (e.g., birth asphyxia, congenital central nervous system (CNS) infections, CNS injuries, tuberous sclerosis complex, and metabolic disorders) [1]. Cryptogenic causes of LGS are increasingly linked to genetic disorders such as chromosomal syndromes and de novo mutations [2]. Among patients with seizure-related disorders connected to mitochondrial diseases, the diagnosis of LGS is as high as 25%. However, mitochondrial dysfunction remains an extremely rare cause of LGS [4,5]. About 20% of LGS cases are preceded by a diagnosis of West syndrome/infantile spasms [1]. Such progression is linked to poor seizure and neurologic prognosis [2]. As many LGS patients develop pharmacoresistance, several non-pharmacological treatment options are used, including surgical and dietary methods [6]. Tonic seizures are considered the most drug-resistant, while atypical absence seizure and myoclonic seizure pharmacotherapy is more effective [7]. 

## 2. Review Method

In this review, we intended to present literature reports about the effectiveness of major non-pharmacological therapies used in the treatment of LGS. We performed a literature search on the PubMed database using the following MeSH terms: “Lennox Gastaut Syndrome”, “Deep Brain Stimulation”, “Vagus Nerve Stimulation”, “Hemispherectomy”, “Diet, Ketogenic”, and terms not indexed in MeSH: “Responsive Neurostimulation”, “Resective Surgery”, and “Callosotomy”. 

We started with 261 records. Twelve papers were excluded due to non-English language. We focused on papers describing therapeutic techniques, patient qualification, safety, and efficacy. We preferred studies with quantitative and comparable data on seizure reduction. Ultimately, we collected 100 papers published between 1990 and 2025. We also added two records from the UpToDate website to provide more general information on epilepsy and its surgical treatment.

Outcomes were assessed regarding the proportion of responders (usually described as patients achieving ≥50% seizure reduction rate) and seizure-free patients. In studies on resective surgery, we used the proportion of patients achieving Engel classes I-IV instead, as it was the most common way to evaluate post-operative seizure reduction.

## 3. Surgical Therapies

Resective surgery methods, corpus callosotomy (CC), and vagus nerve stimulation (VNS) are major surgical methods in LGS [3,7]. If resective surgery cannot be performed due to the inability to detect the seizure-onset zone, VNS, DBS, RNS and CC are considered palliative methods that can provide effective seizure control [8].

In order to localize the seizure-onset zone and save the functional areas of the cerebral cortex, a precise surgical evaluation is performed before surgery [9]. If the focal epileptogenic zone can be detected, resective surgery should be offered to the patient [7,10]. Diagnostic methods comprise non-invasive ones, such as positron emission tomography (PET), single-photon emission computed tomography (SPECT), and magnetoencephalography (MEG) [7,9]. When non-invasive methods fail to localize epileptogenic foci, invasive methods, such as long-term intracranial electroencephalography (EEG) monitoring (icEEG) and stereoencephalography (SEEG), are considered. In two different studies, the major complication rate of icEEG was reported as 6.6% [11] and 9% [12], respectively (mostly infections and hemorrhages) [11]. The use of subdural electrodes was linked to a higher risk, compared with depth electrodes [12]. Compared with icEEG recording, the efficacy and safety of SEEG are considered favorable [9], with a pooled complication rate of 1.3% [13]. 

However, up to 40% of patients who underwent SEEG are not offered surgery, due to the inability to identify a seizure-onset zone, or its less-focal nature [14]. In order to predict situations where SEEG is unlikely to find a focal seizure-onset zone and reduce unnecessary invasive procedures, Astner-Rohracher et al. [14] designed a 5-SENSE score calculator (5-SENSE), consisting of the following variables: “focal lesion on structural magnetic resonance imaging (MRI), absence of bilateral independent spikes in scalp EEG, localizing neuropsychological deficit, strongly localizing semiology, and regional ictal scalp EEG onset”. During score development analysis carried out among 128 patients, specificity and sensitivity were 76.3% and 83.3%, respectively. Validation including 207 patients showed that specificity and sensitivity were 76.0% and 52.3%, respectively. According to the authors, the 5-SENSE may be a simple and useful tool for clinicians, due to high specificity [14]. The tool is available online at https://lab-frauscher.Github.io/Sense_calc/ (accessed on 2 April 2025).

Other diagnostic methods that could be useful for the surgical treatment selection are based on the causal connectivity of the brain, including analyzing EEG outcomes via multivariate autoregressive modeling (MVAR) methods [15]. In a study investigating the significance of causal connectivity in detecting epileptogenic areas, Hur et al. [15] demonstrated that direct directed transfer function (dDTF)-based MVAR combined with other neuroimaging methods could be a beneficial tool in detecting primary epileptogenic areas.

### 3.1. Resective Surgery

As mentioned before, resective surgery is the preferred option when primary epileptogenic areas can be localized using diagnostic methods [7]. It can provide immediate and spectacular results in seizure control [3] and targets a wide range of seizure types [16]. However, resective surgery is rarely an option for LGS patients, as they often have multifocal or diffuse CNS abnormalities [1]. Here, we present studies regarding the use of resective surgery techniques among LGS patients.

Kang et al. [17] retrospectively investigated the long-term efficacy of resective surgery among LGS patients, showing that half of them achieved seizure-free status. Patients who underwent surgery within five years of seizure onset were more likely to remain seizure-free, compared with patients who underwent surgery at least 10 years after seizure onset (62.5% vs. 37.5%, respectively). Among resective surgery methods, hemispherectomy showed the greatest effectiveness in seizure control. Additionally, the authors showed that the social quotient (SQ) level was significantly higher among seizure-free patients compared with seizure-resistant patients at the first (from two to three years) and the second follow-up (from four to six years). Similarly to reaching seizure-free status, the presurgical SQ level was also inversely correlated with the time between seizure onset and surgery. What is more, the authors stated that generalized slow spike-and-wave (GSSW) EEG pattern and focal epileptiform discharges were more frequent in the seizure-persistent group [17].

In another retrospective study, Liu et al. [18] studied the outcomes of different resective surgical methods, including single-lobe resection, multilobar resection, partial CC, and multiple subpial transection (MST) in various combinations. Similarly to previous studies concerning surgical treatment of LGS, the authors reported significant seizure reduction with 38.9% seizure-free outcomes. In the study, an improvement in expressive language function, EEG pattern, memory, quality of life (QOL), and cognitive function was also seen along with the mean intelligence quotient (IQ) increase from 56.1 ± 8.1 to 67.4 ± 8.2 after surgery. The results also suggest that IQ improvement is inversely correlated with the age of patients at the time of surgery and with the time interval between the onset of the seizure and the surgery [18].

In a similar study conducted by Lee et al. [19], most LGS patients became seizure-free, and most of the 20 Engel I and II class patients showed improvement in baseline activity, with EEG normalization in five of them. In addition, a correlation was found between the reduction in the number of seizures and improved development, as measured by developmental quotient (DQ) scores used by a psychologist. Among 72.7% of the patients, an increase in DQ score was seen after the surgery, including 27.7% of the patients with a significant improvement (DQ score increased by at least 15 points). In addition, 77.8% of patients improved their scores on the global assessment of development measured by parents or caregivers. On the other hand, a decrease in global development scores was observed in 7.4% of patients [19]. 

The detailed data on methods of resective surgery are presented in Table 1.

### 3.2. Corpus Callosotomy

CC is the most popular method of palliative surgery, preventing the propagation of epileptic discharges between cerebral hemispheres [6,23]. Its first description was made in 1940, and since then, the procedure has undergone several modifications, including different surgical techniques and radiosurgery methods [23].

Regarding the range of resection, it can be divided into total CC and partial CC. A traditional open CC aims for two-thirds of the corpus callosum, sparing the isthmus and splenium in order to reduce the risk of disconnection syndrome [24]. Although the total CC is more effective [25,26], the anterior CC is often preferred as it disconnects the majority of corpus callosum fibers and has fewer complications than the total CC [25]. Moreover, for the anterior CC non-responders, a two-stage total CC can be considered, which seems safer than one-stage total CC [27,28]. Literature data suggest that, compared with adulthood, childhood is a more beneficial time to execute CC [26]. CC was suggested to be most effective in atonic, atypical absence, and generalized tonic-clonic seizures (GTCS) seizures, with lower efficacy in tonic and myoclonic seizures. It was also suggested as a valuable option for seizure types that are remarkably predisposing to patient morbidity, such as drop seizures [16]. 

CC is generally well tolerated [29]. Its typical complications are acute disconnection syndrome, transient weakness, hemiparesis, and aphasia [29,30]. However, the symptoms are rarely persistent [29].

Regarding how the corpus callosum is accessed, CC techniques can be divided into several groups.

#### 3.2.1. Open Surgical Corpus Callosotomy Methods

Traditional open CC requires a craniotomy with a rectangular incision made along with the location of the corpus callosum [29]. Endoscopic CC may be a valuable alternative, allowing for minimization of the craniotomy size. However, due to greater dependence on patient anatomy and postoperative adhesions, its use may be limited [29]. Here, we present data from studies on open CC methods among LGS patients.

In a prospective study, Liang et al. [25] evaluated the efficacy of anterior CC and pharmacological treatment in school-age patients with LGS. Participants were divided into a pharmacological and a surgical group. In this study, seizure-free outcomes were more frequently achieved in the surgical group. In the surgical group, the greatest reduction in seizures was achieved in tonic seizures, with the smallest reduction in myoclonic seizures. Similar to the efficacy in seizure reduction, surgery outperformed antiseizure medications (ASMs) in IQ improvement (43.5% vs. 5.9%, respectively). Significant differences were also found in overall QOL. Ding et al. [20], in a similar prospective study, compared the efficacy of surgical and multi-ASM therapies in school-age patients with LGS. Similarly to the previous study, the participants were divided into a pharmacological group and a surgical group, including the subgroup of resective methods and the subgroup of combined resective methods (resective methods combined with anterior CC). The surgical group consisted of patients with localized epileptogenic zones, with no such patients in the pharmacological group. It was found that in the surgery group, the seizure-free outcome ratio was significantly higher (69.8% vs. 4.0% at first-year follow-up, 60.5% vs. 0.0% at the third-year follow-up, and 54.5% vs. 0.0% at the fifth-year follow-up, respectively). No significant differences in seizure reduction were detected among surgical subgroups. Moreover, in subsequent follow-up examinations in both subgroups, the percentage of seizure-free patients gradually decreased. Assessment of improvement in IQ and memory quotient (MQ) in the third-year follow-up showed significantly better results in patients who underwent surgery (67.4% and 37.2% compared with 8.7% and 4.3%, respectively). QOL improvement showed a similar tendency (72.1% in the surgical group and 4.3% in the pharmacological group). Furthermore, there was a significant advantage of resective surgery combined with anterior CC over resective surgery alone in overall IQ and QOL changes. On the other hand, no significant difference was found in the changes in MQ. Adverse effects of treatment were described as transient and occurred within three weeks. The authors suggest that resective surgery combined with CC may be a beneficial therapy for LGS patients in terms of significant reduction in seizures, as well as IQ and QOL improvement among LGS patients with the localized epileptic zone [20].

Kurwale et al. [31] investigated the use of resective surgery and CC among 38 patients with drug-resistant epilepsy (DRE) syndromes “secondary to posterior cortex ulegyria as a sequeale of perinatal insults”, i.e., hypoxic ischemic encephalopathy (HIE) or neonatal hypoglycemic brain injury (NHBI). The study population included 18 LGS patients who were administered microscopic complete CC, of whom 10 had a documented history of HIE/NHBI. All the patients experienced multiple seizure types, including disabling drop attacks, and 10 of them had at least severe intellectual disability. The surgery ceased the disabling drop attacks in all patients, with noticeable improvement in EEG results and reduced need for ASMs. In the postoperative period, four of 18 patients demonstrated symptoms suggesting acute disconnection syndrome (i.e., confusion, apathy, and increased response time latency), but the symptoms “improved to pre-surgical level by the time of discharge”. Moreover, 12 of 18 patients showed improved eye contact, improvement in social interactions, behavior, and attention. Another study included nine LGS cases in patients with DRE, but there were no data available on individual LGS patients in the corresponding resective surgery group [31]. 

Katagiri et al. [8] investigated the efficacy of another combination of surgical therapies—CC with subsequent VNS in patients with LGS. In their study, they found that CC provided an excellent reduction in drop attacks in the majority of patients with drop attacks, whereas when considering all other seizure types, only a few patients (20%) were defined as responders. No seizure freedom was achieved, and half of the non-responders experienced seizure exacerbation. Subsequent VNS was effective for all seizure types (except for drop attacks) in 60% of the patients, including 50% of the patients who were classified as CC non-responders. Additionally, the authors showed that conversation ability before the VNS was significantly correlated with achieving responder status [8].

In another prospective study, the comparison of CC and VNS was conducted by Cukiert et al. [32] among LGS and LGS-like patients. The participants were divided into two groups: Group 1 was treated with anterior-half CC and Group 2 was treated with VNS. At the two-year follow-up, 10% of Group 1 patients achieved seizure freedom, whereas in Group 2, no such cases were recorded. The rate of non-responders was also higher in Group 2 (16% vs. 10%, respectively). Both CC and VNS were effective in reducing the frequency of atypical atonic seizures and GTCS, but neither was effective for tonic seizures. CC was particularly effective in reducing the frequency of atonic seizures, while VNS was ineffective. Conversely, CC was not effective in reducing the frequency of myoclonic seizures, while VNS was. Rupture of secondary bilateral synchrony was shown in 85% of CC patients and none of the VNS patients. Improvement in attention and QOL was seen in 85% of patients from each group [32]. Group 2 patients needed a shorter hospitalization after surgery (a mean of 2.0 vs. 7.5 days, respectively). The authors stated that, according to the adverse effect panel, VNS may be a good initial approach, whereas CC could be preferred in cases of predominant atonic seizures [32].

#### 3.2.2. Minimally Invasive Corpus Callosotomy Methods

A traditional open CC requires entrance to the corpus callosum, which carries a risk of severe complications caused by nearby structure damage, including intracranial hemorrhage, lower extremity weakness, and sinus thrombosis. Another complication associated with surgery is infection of the surgical site [24]. Importantly, the fear of such complications is the main reason for the reluctance to undergo brain surgery among patients with epilepsy and their families [33].

A stereotactic laser anterior CC (SLACC) has been designed as a minimally invasive alternative to surgical CC. It allows for reducing side effects, as well as the time of hospitalization after the procedure [34]. In a report of two cases, Tao et al. [35] demonstrated that the SLACC procedure yielded beneficial results in both patients included in the study. Each had previously been treated with VNS and both had taken four ASMs, without achieving a satisfactory reduction in seizures. After SLACC, both patients showed notable improvement in clinical outcomes, including a reduction in the frequency of generalized paroxysmal fast activity (GPFA) and improved attention and verbal expression. One patient remained seizure-free during 18 months of follow-up, and the other achieved more than 90% seizure remission during seven months of follow-up. The first patient showed transient postoperative hypersomnia and remained in the hospital for four days. The second patient was discharged after two days without complications. The authors suggested that SLACC could be an effective and potentially safe alternative to open CC [35].

Another retrospective study to assess the efficacy and safety of SLACC was conducted on 10 LGS patients [24]. Nine had previously undergone VNS without satisfactory clinical improvement. Four patients had been offered an open CC in the past, but no CC was performed due to a lack of consent from legal guardians. The median hospital stay was one day (ranging from one to four days). Furthermore, the authors demonstrated a modest reduction in interictal discharges and a marked reduction in ictal discharges in nine patients. According to the authors, SLACC had an efficacy comparable to the reported efficacy of open CC and was well tolerated [24]. Rich et al. [28] studied the efficacy of different variants of stereotactic laser corpus callosotomy (SLCC) in a population of 13 adult epilepsy patients, of whom 9 had LGS. Most patients had used VNS, however, with unsatisfactory results. In total, 15 SLCC procedures were performed, including 10 SLACC, 1 complete SLCC, 2 posterior SLCC (SLPCC) following prior SLACC, and 2 SLPCC following prior open anterior CC. The mean follow-up time was 20 months. At the last follow-up, 10 of 13 patients had at least a 50% reduction in seizures, and the remaining 3 patients had not worsened [28]. In a recent meta-analysis of the general epilepsy population, Awad et al. [36] suggested that laser CC is comparably effective as a traditional open CC in providing seizure reduction. Moreover, its use may result in patients experiencing fewer adverse effects and shorter hospitalization time [36].

Another less-invasive CC method is based on radiosurgery. Pioneers of radiosurgical CC, Gerhard Pendl et al. [37], in a study published in 1999, described the cases of three patients with intractable epilepsy syndromes, including two patients with LGS [37]. In the study, a cobalt-60 Gamma Knife using a 4 mm collimator was used to ablate the anterior third of the corpus callosum. Following the procedure, all three patients experienced significant reductions in seizure frequency and severity, particularly in atonic seizures and GTCS. However, the psychosocial and cognitive functions of the patients did not improve. The pioneers of this method suggested that radiosurgical CC may be a beneficial alternative to traditional open CC [37]. Another case series by Eder et al. [38] showed less beneficial results. In three patients, one of whom was a patient with LGS after anterior CC, the seizure frequency did not change. The only benefit in this patient’s case was a reduction in aggressive behavior. On the other hand, both remaining epilepsy patients showed a 100% reduction in GTCS and an improved mental and physical condition [38]. Another case of radiosurgical CC was reported by Sachdev et al. [39]. The study described a case of a 20-year-old male patient who underwent a complete CC by stereotactic radiosurgery (SRS) using a Gamma Knife. Notably, the patient had previously undergone an anterior two-thirds CC at age 10, which resulted in marked improvement, but two years prior to the SRS procedure, a gradual deterioration in both behavior and seizure control was observed. The biggest problem was the recurrence of atonic seizures, as a result of which the patient suffered multiple injuries. The SRS procedure resulted in marked improvement in behavior and seizure control, including the absence of atonic seizures and improvement in other seizure types. The evaluation of clinical features performed at the third and eighth month after SRS showed that the patient’s improvement was still maintained. The authors stated that SRS complete CC may be an effective option for refractory seizure control [39].

Another interesting CC procedure was presented by Baumgartner et al. [40]. They described a case of a five-year-old LGS patient with a unique anatomy. Due to the ventriculomegaly and thinning of the cortex and corpus callosum, surgical CC and laser CC posed a danger of ventricular collapse and hemorrhage. Thus, the authors performed CC with a transventricular “inside-out” approach—an approach not achievable in normal anatomy. They used a single endoscopic working channel inserted via a 7 mm burr hole left after a past ventriculostomy. Complete CC was performed without intraoperative complications, and the patient achieved seizure reduction with an increase in quality of life [40].

The promising safety profile and the results comparable to microsurgical CC suggest that SRS CC could be an attractive option for CC, and it could be used complementary to VNS or for complete CC after microsurgical anterior CC. On the other hand, there is not enough literature on SRS CC to prove its efficacy, and further randomized controlled trials (RCTs) are still needed to evaluate its efficacy and safety.

The detailed data on CC are presented in Table 2.

### 3.3. Neuromodulation Therapies

The three major neuromodulation therapies are VNS, deep brain stimulation (DBS), and responsive neurostimulation (RNS), of which DBS constitutes the most promising approach for LGS patients. These modalities, however, require optimization of stimulation locations and paradigms, as well as development of reliable biomarkers of seizure burden and comparative studies of efficacy providing evidence for appropriate treatment selection [43]. They are an option for patients with DRE not eligible for resective surgery [44,45] or patients who present unsatisfying seizure reduction post-surgically [45], and their off-label use for LGS patients is currently increasing. This population includes patients with multifocal seizure onset [45], or with epileptic foci within an eloquent cortex [44,45], or with unknown localization [44].

#### 3.3.1. Vagus Nerve Stimulation

VNS was approved by the Food and Drug Administration (FDA) for the treatment of refractory epilepsy in 1997, although its first use for treating epilepsy dates back to the 19th century. The most commonly used device is the surgically implanted VNS system, although new non-invasive alternatives emerged, such as the transcervical (tcVNS) and transauricular VNS (taVNS). The electrodes of the invasive device are located on the left vagus nerve below the inferior cervical cardiac branch offshoot. The stimulation parameters, such as output current, stimulation time, and current frequency can be adjusted, and the device can be controlled manually using the external component [46], although there are no clear guidelines settled for different seizure types [47]. Recently, Fahoum et al. [48] provided evidence-based guidance for the population-level target of VNS dose with a current intensity of approximately 1.625 mA and a duty cycle of 30 s ON, and 3 min OFF to achieve maximal response in patients with DRE. This study, in contrast to previous guidance presented by the American Academy of Neurology (AAN) in 2013 [49], recommended a higher current output to improve seizure reduction without the risk of increasing adverse effects. However, the authors emphasized possible differences in VNS doses for improving mood or cognitive functions, variability in clinical response in individual patients, and the lack of data to evaluate new stimulation parameters (e.g., high frequency burst stimulation) [48]. In the case of invasive VNS, the most frequent adverse events are related to the implantation and may include superficial infection, vocal cord paresis, cough, neck pain, and lower facial paresis [50]. The most common side effects associated with stimulation in LGS patients are relatively mild and include voice alteration, hoarseness, and salivation, and most of them are transient [30,51]. The rate of seizure reduction of 50% or more in LGS patients [30] is similar to the efficacy in all DRE studies and is approximately 50% [51,52]. VNS therapy can be used in combination with various ASMs (no interaction with ASMs) and non-pharmacological methods, such as diet or other surgical methods [53].

Suller Marti et al. [54] studied retrospectively the efficacy of VNS in reducing the number of seizures in patients with refractory epilepsies such as genetic generalized epilepsy (GGE) and LGS. It was effective, although not significantly, only for GTCS (no effect on atypical absence, typical absence, and myoclonic seizures). However, hospital admissions have fallen significantly, probably due to an overall reduction in seizures. There was no significant improvement in the number of ASMs required, with the median number remaining at three. The side effects related to stimulation occurred in the majority of LGS patients and were largely insignificant. However, the lack of efficacy in seven patients (24.1%) led to the discontinuation of the VNS device. The authors suggested that VNS should be considered in patients with generalized DREs [54].

Braakman et al. [55] published a case report of a 16-year-old male who had a long and almost sinusoidal treatment history consisting of pharmacotherapy, ketogenic diet therapy (KDT), and VNS (implanted at the age of five). The patient eventually became seizure-free for eight consecutive months, and his cognitive development improved since he first had seizures at 13 months of age. As the authors described this case as “remarkable in several ways”, it provides information on the significant efficacy of VNS in controlling seizures and improving psychomotor performance [46]. 

Cukiert et al. [56] presented a prospective study on the effectiveness of VNS “at maximally tolerated current intensity” in 24 LGS and LGS-like patients. The initial VNS parameters were 0.25 mA, 30 Hz, 500 μs, 30 s ON, and 5 min OFF. Then, the current intensity was being adjusted by 0.25–0.5 mA, depending on its tolerability and efficacy, aimed at a maximum of 3.5 mA. Eventually, the tolerated current intensity stabilized at an average level of 3.1 mA (2.0–3.5 mA). A transient worsening of seizure frequency occurred in 41% of patients, due to increased stimulation current. ASM administration was not changed throughout the observation period. A “honeymoon effect” was observed in 14 patients, defined as a ≥50% reduction in seizure frequency immediately after VNS implantation while the device was still OFF (VNS was turned ON after three weeks or after the “honeymoon effect” resolved), which lasted an average of 20.2 days. Overall, VNS was effective in reducing only a subset of seizure types (effective for atypical absence, GTCS, and myoclonic seizures; ineffective for atonic and tonic seizures). The “honeymoon effect” although elusive and not necessarily significant in the long term, may have an impact on caregivers’ initial perception of the VNS procedure. In addition, investigators observed improvements in QOL and attention levels [47]. 

Narayanan [57] published a case report of four patients with LGS in whom the total daily charge was routinely increased because of a lack of satisfactory response to VNS with standard parameters. A rapid stimulation that consists of a 7 s ON and 30 s OFF pattern can be set to improve seizure control in patients who do not respond effectively to standard stimulation (30 s ON and 300 s OFF) [57]. However, despite possibly good tolerance and efficacy, there is no clear evidence that this method is overall better than the standard one [47,57]. What makes this particular case unusual is the effect of an increase in seizure quantity and severity to almost pre-VNS level in all four patients after switching to a rapid stimulation approach (which followed the initial increase in the output current to 2.00–2.25 mA), and additionally withdrawal of this phenomenon after a return to the previous total charge delivered per day. The increase in seizures was noted within one to two weeks after the stimulation pattern switch, and the same time range concerned the following decrease. The author pointed out the non-convincing evidential value of this case because of the low number of patients and only a single occurrence, which at the same time could not be repeated due to ethical reasons. Nevertheless, it is a worth-mentioning observation [57] that corresponds with a previous finding of worsening in seizure frequency as a result of the current increase [56].

A retrospective review considering the effectiveness of the auto-stimulation VNS devices in 71 LGS patients aged between one to eighteen years was published by Abdelmoity et al. [58], who collected the data from a single epilepsy center. In children, a self-stimulating VNS model was implanted as a new therapeutic method (or as a replacement for the previous non-self-stimulating VNS system), which detects an ictal tachycardia and responds with additional stimulation to terminate the seizure. The initial number of ASMs per patient was 3.4, and there was no significant change over the course of the study. The stimulation parameters followed the standardized VNS protocol to achieve maximum tolerability. Compared to older VNS models, an additional 60–70% of patients using newer models experienced a further reduction in seizure frequency. The study demonstrated VNS models with auto-stimulation, especially with regard to reducing the seizure frequency, and also provides a source of information on the efficacy and good tolerability of VNS in the treatment of LGS [58].

Kostov et al. [59] conducted a retrospective study on the long-term effectiveness of VNS in 30 patients with drug-resistant LGS, focusing primarily on the seizure type and side effects of the therapy. Due to the significantly different impact of various seizure types on activities of daily living, they were divided into “disabling” (GTCS, tonic, and atonic) and “nondisabling” (atypical absences, complex partial [CPS], and myoclonic). The median number of ASMs was reduced from 3.0 to 2.0. The authors found an overall beneficial effect and good tolerability of VNS in patients with LGS and described the therapy as encouraging and very promising for these individuals. It was suggested that VNS may be a valuable alternative therapy for patients with LGS because it correlates with fewer adverse effects compared to polypharmacy and CC. It was also suggested that because of the most significant reduction in the frequency of two of the three “disabling” seizure types, such as tonic and atonic, and the least effect on the third type (GTCS), there is a need for a more selective use of VNS with respect to seizure type. Additionally, the use of magnets by the patients or their relatives could reduce seizure duration and severity [59].

Another retrospective chart review executed by Kayyali et al. [47] addressed the issue of optimizing VNS stimulation parameters by using a rapid duty cycle (RDC) with an OFF time of ≤1.1 min, while maintaining a duty cycle of <50%. The study included 50 patients aged one to 17 years with intractable epilepsy, including LGS, and 44 of them were finally analyzed for outcome. The regular treatment cycle lasted an average of 1.47 years (SD = 1.08) without affecting efficacy, and the mean duration of RDC was 2.99 years (SD = 1.85). Although no data were presented for patients with LGS, the overall responder rate increased from 45.5% before RDC to 77.3% and 77.4% at the first RDC follow-up visit and the last RDC visit, respectively. In addition, 34.1% of patients became responders, and two patients became non-responders after switching to RDC VNS. The authors concluded that RDC VNS was likely to be safe and tolerable, and that it was likely to be superior to regular cycling. However, the investigators indicated the need for further controlled and larger cohort studies on this topic [47].

One of the early studies on the effectiveness, tolerability, and safety of VNS in 50 pediatric patients with LGS was conducted by Frost et al. [60]. In a retrospective review, the researchers primarily assessed the efficacy of the therapy with an additional assessment of adverse effects and QOL. Various other therapeutic methods had been tried before the implantation in some patients, such as ASMs (a median of nine drugs), KDTs (36% of the patients), CC (10%), and lobectomy (2%). The most prevalent seizure type was the drop attack, which also responded best to the therapy, with a median seizure reduction of 88% after six months. Furthermore, atypical absence seizures were significantly decreased with a median reduction rate of 73% and 81% after three and six months, respectively, and in contrast, CPS showed a poor response to the treatment with a median reduction of 23% after three months post-implantation. There was also a 73% and 81% reduction in seizures at three and six months, respectively, in patients who had previously undergone CC, compared to no change in the patient who had undergone lobectomy. No significant difference in treatment response was observed between children under 12 years of age and the entire group. QOL was described as better or unchanged in most patients, with only a few cases of worsening in some fields. When it comes to adverse effects, these were reported either due to the surgery itself (only a few transient events, such as wound infections or localized pain) or the stimulation therapy, which was well tolerated, and 26% of the group reported no stimulation-related adverse effects at all. The typical device settings were 1.25 mA, 30 Hz, 500 μs, 30 s ON, and 5 or 10 min OFF. The stimulation changes (including RDC) did not appear to enhance efficacy, while stimulation duration was possibly an important factor. 

Aldenkamp et al. [61] analyzed the long-term (24 months) effectiveness of VNS as well as its impact on QOL and cognitive functions in 17 patients with LGS or LGS-like syndromes. The researchers evaluated the behavioral effects of VNS using various scales assessing cognitive function (mental age) and QOL (independence, behavior, and mood). During the study, no significant changes in administration of ASM were observed, which, in most cases, consisted of polytherapy with two to four drugs. The authors looked for a correlation between mental age and the three aspects of QOL over time, which was ultimately not found to be significant in any combination. The most consistent change was an increase in mental health of 4.2 months over a 24-month period. Although statistically insignificant, a relationship was suggested between the overall reduction in seizure frequency and the change in mental age, and the highest improvement in cognitive function was noted in the responder group. It was concluded that “VNS does not have any adverse effects on higher-order functions or QOL, even in the long term”, and that “mental retardation can be characterized as a negative prognostic factor for treatment with VNS”, given that the patients with the highest level of mental function responded best to the therapy. The authors also noted a positive, although mild, effect on mental age in patients without a reduction in seizures, which, however, was not stable over time, probably due to the specific group of patients with high mental retardation. Therefore, it was also suggested to test the direct effects of the VNS therapy on behavior, but this time in children without mental disabilities [61].

Another retrospective study on the effectiveness, tolerability, and safety of VNS was conducted by Cersósimo et al. [62]. The group of patients consisted of 64 children with refractory epilepsies, including 46 with LGS. The LGS patients had multiple seizures (except for one case), all had mental retardation, which was severe in 17 cases, and seven children had undergone a clinically ineffective CC before the VNS. Prior to the VNS therapy, all patients were given at least two ASMs (all unsuccessfully). The therapy resulted in a seizure reduction of 80% or more in 28 patients (60.9% of the LGS group), 50–79% in 12 children (26.1%), 6 patients had a reduction of under 50%, and none of the children achieved seizure freedom. The number of responders in the LGS group was 30, and all LGS patients showed improvement in ictal or postictal severity of seizures. By seizure type, the greatest reduction was observed for tonic seizures and drop attacks, whereas atypical absences showed a poorer response, and the smallest reduction was evident for focal seizures and epileptic spasms. Neither the reduction in seizure frequency nor the severity of mental retardation differed between cryptogenic or symptomatic patients, and efficacy was the same regardless of whether the children previously suffered from West syndrome or not. Furthermore, patients who underwent CC prior to VNS had no better seizure frequency outcomes than patients who did not have a prior history of CC. The authors reported an impact on QOL in all 64 patients, with almost all (except three children) showing significant improvement in behavior and cognitive function, and a good response to VNS was always accompanied by an improvement in mental age. The number of ASMs decreased in 20.3% of 64 patients (no separate data for LGS patients). The initial marked improvement in efficacy continued throughout the study. A direct positive effect of VNS on QOL has also been suggested, which might be independent of the reduction in the number of seizures [62].

The researchers claimed that “VNS is an effective treatment for patients with refractory epileptic encephalopathies and focal epilepsies and effectiveness is also shown for different types of epileptic seizures” [62]. VNS was also suggested to be particularly effective in atonic and tonic seizures, while data on GTCS and absence seizures remain inconsistent [16]. It is suggested that VNS is appropriate in patients with LGS and recommended before CC because it is associated with fewer serious side effects and better efficacy [60].

Both the implantation and stimulation procedures are generally well tolerated [62,63]. Of note, to make VNS more convenient and safe, some improvements emerge in VNS technique. The closed-loop, miniaturized, wirelessly powered device described by Mathews et al. [64] could serve as a promising example for the VNS future.

#### 3.3.2. Deep Brain Stimulation

DBS is an emerging DRE therapy [65]. It relies on the implantation of electrodes into subcortical central nervous structures [65]. Before it was implemented in the treatment of DRE, it was primarily employed as treatment for movement disorders, such as Parkinson’s disease. Neurostimulation of many structures was shown to provide seizure control; however, in the treatment of DRE, the two most common targets are the anterior nucleus of the thalamus (ANT) and the centromedian nucleus of the thalamus (CMT) [44]. ANT DBS gained FDA approval for the adult DRE population [66] and was suggested as a promising target in younger LGS patients [67]. To target ANT more precisely on MRI, fast gray matter acquisition T1 inversion recovery (FGATIR) was suggested to allow localizing ANT using mammillothalamic tract visualization [68]. On the other hand, it was suggested that DBS of the CMT (especially the anterior and inferolateral border of its “parvocellular” component [65]) is particularly effective in LGS patients [68], and CMT is recently considered the main target for such patients [16]. 

One of the milestone studies on DBS in LGS patients was an RCT titled “Electrical stimulation of thalamus for epilepsy of Lennox–Gastaut phenotype (ESTEL)” by Dalic et al. [69], who investigated the efficacy and safety of continuous, cycling stimulation to the bilateral CMT in 19 young adults with LGS, of whom 10 were randomized into the treatment group. After three months, the non-significant diary-recorded seizure reduction of ≥50% was achieved in 50% of the stimulation group, compared with 22% of the control group (*p* = 0.25). However, a significant effect was noted for electrographic seizures, where 89% of the treatment group had a ≥50% reduction at the end of the three-month blinded phase, compared to none in the control group (*p* = 0.05). The authors thus highlighted the need for applying objective markers of seizure frequency, given the limitations of diary-recorded seizure counts, which, despite the best efforts of caregivers, might be capturing a small fraction of electrographic seizures. Additionally, the researchers pointed out that a relatively low maximum stimulation delivery of 2.5 V might have contributed to worse outcomes regarding the efficacy of reduction in seizures (thus highlighting the need for establishing the optimal stimulation paradigms for DBS in LGS), and that the stimulation of other thalamic nuclei might be similarly or more effective. The treatment was overall well-tolerated. The caregivers of nearly all subjects reported an increase in alertness, and the majority reported overall benefit. Due to possible cellular, molecular, and neuroplastic changes in pathological brain circuits that are occurring over time after the DBS treatment, a continued follow-up of the ESTEL trial cohort was stated as crucial in the assessment of CMT DBS efficacy [69], which is expected in late 2025 with 5-year follow-up data [43]. Given the above-mentioned under-reporting of seizure frequencies in diaries, the cohort of 17 subjects from the ESTEL trial was also analyzed by Dalic et al. [70] with the aim of identifying a fast, reliable, and objective biomarker of treatment response. With the use of intermittent, 24 h EEGs, the authors evaluated GPFA, which is an easily identifiable primary interictal electrographic feature of LGS. Baseline daily median seizures recorded in diaries were 2.6 (interquartile range (IQR) = 1.4–5), while the electrographic seizures were 284 (IQR 120.5–360) per day. The researchers found a strong association between changes in GPFA count and changes in diary-recorded seizures in individual patients. The study showed that GPFA might be a usable biomarker of response to therapy, allowing a rapid titration of treatment parameters, as opposed to the time-consuming and possibly inaccurate seizure diaries [70]. 

In the earlier work from 2006, CMT DBS was used in 13 LGS patients with severe GTCS and atypical absence seizures. The implantation of DBS provided an overall 80% seizure reduction, with two cases of seizure freedom. After the procedure, the group as a whole showed significant improvement in ability, with two of the patients “living a normal life” at the end of their follow-up. The additional beneficial effect of the DBS was improvement in the QOL [71]. 

Poulen et al. [67] documented three cases of adult LGS patients treated with ANT DBS. All patients achieved significant seizure control with at least 75% seizure reduction in each subject patient. Moreover, the patients presented marked improvement in adaptive behavior [67]. In the recent meta-analysis, DBS showed a pooled 50% responder rate at 69.7% and outperformed other neuromodulation modalities [72]. It presumably controls GTCS, atypical absence, and tonic seizures most effectively, with less beneficial effects on focal seizures [16]. Late onset of epilepsy was linked to the beneficial response to DBS [72].

Compared to VNS, the safety of DBS is less often reported [72]. Many studies documented no adverse effects or no postoperative complications [67,73]. In the recent meta-analysis of RCTs, Zhu et al. [74] suggested that DBS is less prone to cause adverse effects than several ASMs. On the other hand, there are reports on complications requiring device explanation, i.e., adverse skin reactions, and electrode rupture. Some mentioned disadvantages of VNS include the following: a need for temporary battery replacement and a need for temporary confirmation of correct functioning [71]. Neidhart et al. [75] described the case of the LGS patient with CMT DBS-induced dysarthria and ataxia. However, the symptoms were relieved after the slight reduction in the lateral and superior stimulation, further delineating the value of precision in tailoring the neurostimulation to neuroanatomy [75].

#### 3.3.3. Responsive Neurostimulation

RNS is characterized by its adaptive (closed-loop) nature, in contrast to the continuous (open-loop) stimulating provided by VNS and DBS [44]. Its feature is particularly beneficial if the epileptic focus is located in the eloquent cortex, as its continuous stimulation could disrupt vital essential functions [44]. Adaptive mode also requires less electrical stimulation compared to open-loop systems, which implies longer battery lifespan [76]. RNS is approved by the FDA for the treatment of DRE in adult patients [77] and is more commonly used off-label in the pediatric population [76,78]. It also allows for enhancing seizure control by modifying lead placement or stimulation settings, conversely to irreversible resective surgery [76]. The data on its seizure-specified efficacy are limited and require further investigation [16].

In DRE patients, RNS was suggested to provide continuous improvement in seizure control within several years of its use [76,77]. However, as it is a novel therapeutic method, the literature on its use in LGS patients is limited [44], with studies including up to 10 LGS patients [72]. Novel meta-analysis of neurostimulation modalities in LGS patients by Samanta et al. [72] included 27 patients treated with RNS, with 63% of them reaching at least 50% seizure reduction. 

There are few studies on RNS in LGS patients, most of them being case series of DRE patients. One of the largest was published by Ahn et al. [78]. They included information on 10 pediatric LGS patients treated with bilateral CMT RNS. The majority of patients presented a beneficial response to the RNS. Of note, the authors also linked higher response rates with longer duration of stimulation, with a similar link noted in meta-analysis by Samanta et al. [72] (both not statistically significant) [78].

Another case series of RNS in LGS was performed by Roa et al. [79]. They described their experience with temporal RNS in 23 DRE patients. The majority of LGS patients (and about 65% of DRE population) achieved at least a 50% reduction rate. The authors also noted that epileptic zones located in the temporal lobe were a predictor of better response to RNS [79]. 

In their study on DRE patients, Beaudreault et al. [80] documented the effects of concomitant use of RNS and VNS. The noticed seizure reduction reached a 75–99% range in each of the LGS patients. Beaudreault et al. [81] also prepared another study on RNS, but as no adequate data were provided for LGS patients, we were unable to assess seizure reduction rates. However, each of the four LGS patients included in this study achieved improvement in seizure severity, seizure duration, or both [81]. Fields et al. [45] performed another case series comprising LGS patients, but did not specify seizure outcomes in this subgroup.

Kwon et al. [76] presented two case series describing off-label use of RNS in two pediatric LGS patients—a 16-year-old girl and a 12-year-old boy with rapid, frontotemporal onset of epileptiform activity. After RNS placement, the authors noted that the efficacy and safety were comparable to experiences with adult patients. The caregivers reported further advantages of improved alertness and interactivity, and no surgical complications were documented. The patients were offered RNS with electrodes in CMT. Of note, in the first patient, one of the two electrodes was initially placed in the frontal cortex. The researchers temporarily turned it off, and after they noticed no change in seizure rate, it was placed in the contralateral CMT with further improvement in seizure control. According to them, this further supports evidence on the importance of CMT in seizure pathogenesis in LGS [76]. 

The literature on RNS safety of LGS patients is limited, with some studies not including its minor adverse effects [72]. Kwon et al. [76] reported no adverse effects in the two studied LGS cases. RNS also seems not to be associated with mood or cognitive adverse effects [82]. Zuckerman et al. [83] described a case of 17-year-old old female patient, who developed symptoms of myasthenia gravis five months after RNS placement; however, they claimed its causative role as “extremely unlikely”. In the general DRE population, the most commonly reported complications are related to stimulation or infections. A two-year follow-up of 191 adult patients by Heck et al. [84] resulted in 19 serious adverse effects related to the implanted device, including seven cases of implant site infection, seven cases of lead revision, and five cases of lead damage. Ahn et al. [78] reported stimulation-related side effects in 8 of 16 DRE patients (e.g., painful shocks, dystonic posturing), which persisted in 3 of them. Interestingly, in two of eight cases, stimulation-related symptoms were alleviated by the change from high-frequency stimulation to low-frequency stimulation. They also reported three cases of superficial pocket infection, followed by RNS removal. Of note, a modified perioperative protocol including pre- and postoperative vancomycin resulted in the lack of new infection complications [78]. Similarly, infections were the most common adverse events in the case series by Roa et al. [79]. 

The detailed data on neurostimulation therapies are presented in Table 3.

## 4. Ketogenic Dietary Therapies (KDTs)

KDTs, including the classic ketogenic diet (cKD) and its variants are considered a well-tolerated and effective alternative treatment for DRE [85,86,87,88,89]. The history of diet therapies in the management of epilepsy goes back to the early 1920s [90]. In general, KDTs are based on high fat intake, low carbohydrate intake, and adequate protein intake, putting the organism into a state of ketosis [86,88]. An important parameter of KDTs is the ketogenic ratio, i.e., the mass ratio of fat to carbohydrates plus protein [88]. There are four major KDTs: cKD, medium chain triglyceride diet (MCT), modified Atkins diet (MAD), and low glycemic index treatment (LGIT) [88]. The literature indicates their effectiveness both in patients with cryptogenic causes and in those with structural epilepsy [1] and in a wide range of seizure types [16]. Apart from DRE, it is utilized in Dravet syndrome, infantile spasms, myoclonic-astatic epilepsy, febrile infection-related epilepsy syndrome (FIRES), and myoclonic status in non-progressive encephalopathy (MSNPE) [91]. The strictness and composition of macronutrients in KDTs vary, resulting in different tolerability profiles and practicality [88,92] (see Table 4).

### 4.1. Classic Ketogenic Diet

cKD is considered to be a well-tolerated and effective option for patients with LGS [88]. It is also the most studied and oldest of the KDTs [88,90].

Zhang et al. [93] investigated the efficacy of cKD in the therapy of LGS. The study was conducted among 47 pediatric patients diagnosed with LGS, each of whom had a history of taking at least two ASMs without seizure control. The most commonly used ASMs were valproate, topiramate, and levetiracetam. Thirty-eight percent of the patients had a history of infantile spasms, and 68% of the patients had abnormal MRI scans. During therapy, all patients used their ASMs normally, no changes were allowed.

Seven patients discontinued the therapy between the third and sixth month due to lack of effectiveness and inability to adhere to cKD. However, two of them achieved at least a 50% reduction in seizure frequency. Statistical analysis performed after three months of treatment showed that there was an association between at least a 50% reduction in seizure frequency and the chance of improving the EEG background or reducing interictal epileptic discharges [93].

Na et al. [5], in their retrospective study, investigated the efficacy of cKD and the MAD in the therapy of LGS associated with mitochondrial dysfunction. During the study, four patients from the 4:1 cKD group crossed over to the 3:1 cKD group due to intolerance to the 4:1 cKD. The adverse effects of the therapy were reported in 14 of the patients. No life-threatening metabolic crises were shown. Moreover, in the third month of evaluation, 45% of patients showed improvement in their EEG status. According to the authors, their results suggest that KDTs can be used to treat LGS associated with mitochondrial dysfunction, with results that are non-inferior to those obtained in LGS of other etiologies [5].

Shah et al. presented a different perspective [87]. The authors investigated how often patients with diverse types of DRE were able to achieve drug-free diet (DFD) status with cKD administration. The study included 232 pediatric patients, 48 of whom were diagnosed with LGS. Other most frequently reported epilepsy syndromes were infantile spasms (*n* = 48), epilepsy with myoclonic-atonic seizures (also known as Doose syndrome, *n* = 18), Dravet syndrome (*n* = 14), glucose transporter 1 (GLUT-1) deficiency (*n* = 7), Rett syndrome (*n* = 4), and pyruvate dehydrogenase deficiency (*n* = 3). At the onset of the study, the mean number of ASMs was 2.4 (ranging from 1 to 6), with the most popular being levetiracetam (*n* = 132), valproate (*n* = 71), and clobazam (*n* = 57). After three months of treatment, 70% of 232 patients had a greater than 50% reduction in seizure frequency, and 20% of patients were seizure-free. In 43 patients, DFD status was achieved after a mean of 7.5 months (range of 1.5–21 months). Among the patients who achieved DFD status, 63% were seizure-free, 28% achieved 90–99% seizure reduction, and 9% achieved 50–90% seizure reduction. Of the patients who achieved DFD status, 32 continued on DFD treatment (for a mean duration of 22.6 months), and 11 of these resumed ASMs after a mean of seven months. Factors associated with achieving DFD status included younger age, fewer ASMs at baseline, diagnosis of Doose syndrome, and diagnosis of GLUT-1 deficiency. A diagnosis of LGS and having a gastrostomy tube were factors associated with a lower likelihood of achieving DFD status [87].

### 4.2. Medium Chain Triglyceride Diet

MCT use is based on the fact that medium chain triglyceride use is associated with greater production of ketone bodies, compared with long chain triglycerides (LCT), which are the main source of fat in cKD. This change allows the patients to reduce their daily fat intake and provides more flexibility in product selection [88].

Rosenthal et al. [94] investigated the efficacy of parenteral MCT in a five-year-old female patient with LGS who had previously been successfully treated with oral MCT administration. Her seizures had been deteriorating prior to the initiation of oral MCT, despite the use of multiple ASMs. Oral MCT significantly improved seizure frequency and alertness, but the diet was discontinued because of pseudobulbar palsy. Before starting intravenous MCT, the patient was lethargic and had fair seizure control, but after MCT administration, the patient showed significant improvement in activeness, alertness, and seizure control over the next 24 h. The authors suggested that intravenous MCT could be a relatively safe and effective short-term method in providing parenteral ketosis for seizure control [94].

### 4.3. Modified Atkins Diet

MAD is a less stringent alternative to cKD that does not restrict protein and fat intake and includes nuts, seeds, fruit, and dairy products [3,95]. The ketogenic ratio is not determined, but patients usually achieve the ketogenic ratio of 1:1–1.5:1 [88].

Wu et al. [86] in their study aimed to investigate the effectiveness of MAD in the therapy of various types of DRE encephalopathies in 52 children. All patients were treated with at least two ASMs before participating in the study, and all received MAD for at least 12 weeks. Seven of these patients were diagnosed with LGS, of whom two had a family history of epilepsy, one had a family history of febrile seizures, one had a history of craniocerebral trauma, and two had a history of viral encephalitis. Other patients had a diagnosis of infantile spasms (*n* = 38), Dravet syndrome (*n* = 6), and Doose syndrome (*n* = 1). Adverse effects of treatment were described as not serious. After symptomatic treatment, most adverse effects were alleviated without affecting the course of MAD [86].

Sharma et al. [95] studied the effectiveness of MAD in pediatric patients with LGS whose seizures persisted despite the use of at least three ASMs (with a median of six drugs) tried before starting MAD. The most common causes of the syndrome were structural causes (*n* = 9), polymicrogyria (*n* = 6), and neonatal hypoglycemia (*n* = 6). The diet required limiting daily carbohydrate intake to 10 g. There were no protein or calorie restrictions, while the fat intake was encouraged. Patients continued drug therapy for the first three months and then were allowed to reduce the drug dose based on seizure control. Parents of 12 (48%) patients noticed a subjective improvement in their children’s alertness and interaction. The reasons for discontinuing the treatment were unsatisfactory effectiveness and the feeling that the diet was too restrictive. Reported adverse effects were described as mild and not related to treatment discontinuation in any patient. The authors suggest that MAD is an effective treatment option and may be an ideal choice in the presence of a shortage of trained dieticians due to its ease of administration and good tolerability [95].

### 4.4. Low Glycemic Index Treatment 

LGIT is based on the hypothesis that stable glucose levels play a key role in the mechanism of KDTs. It allows for a daily carbohydrate intake of 40–60 g/day, but prefers products with a glycemic index (GI) lower than 50 [88].

A study conducted by Kim et al. [96] investigated the effectiveness and tolerability of LGIT in DRE therapy. Caloric intake was set at 60% from fat, 30% from protein, and 10% from carbohydrates. Only carbohydrates with a GI of 50 or less were allowed. The diet was described as similar to cKD but less restrictive. The study included 36 patients with DREs, of whom 12 were diagnosed with LGS. Twenty-one patients had received KDTs in the past but discounted them due to poor tolerability. In this study, a good response to previous KDTs was a predictor of good response to LGIT. Two seizure-free patients had a diagnosis of Dravet syndrome and generalized epilepsy, which was not otherwise specified. Two patients discontinued therapy due to a lack of effects and difficulty in adhering to the diet. Either way, the 3-month therapy resulted in a ≥50% reduction in seizure frequency in 20 (56%) patients, and after one year, this effect persisted in 19 (53%) patients. According to the authors, LGIT appears to be an effective alternative for patients with poor cKD tolerability [96].

The data on individual KDTs are shown in Table 5.

## 5. Comparison of Available Treatment Options

Some authors suggest that LGS patients could achieve dramatic improvement in seizure status if they were actively treated multimodally [21,22]. In a large retrospective study, Na et al. [21] showed that a combination of ASMs, KDTs, CC, VNS, and resective surgery techniques could provide a one-year seizure-free status in almost half of LGS patients (*n* = 168 of 371 patients). In this study, patients were actively enrolled in stepwise multimodal treatment. All patients began follow-up with a prescription of at least two ASMs. Forty-one patients achieved a one-year seizure-free status, including fifteen patients who had been seizure-free for at least five years.

Most patients (taking ASMs) with persistent seizures were subsequently prescribed KDTs or surgical treatment, including resective (*n* = 74) or palliative (*n* = 20) approaches. A total of 201 patients were prescribed KDTs, including cKD (*n* = 169), MAD (*n* = 27), and LGIT (*n* = 5). If patients continued to have epileptic seizures, the relevant patients were offered a different procedure. In total, 112 patients underwent resective surgery, including unilobar resection (*n* = 27), multilobar resection (*n* = 60), and hemispherectomy (*n* = 25), and 115 underwent palliative surgery, including CC (*n* = 100) and VNS (*n* = 35). In terms of one-year seizure-free status and five-year seizure-free status, KDTs were about twice as effective as ASMs, and resective surgery and CC led to significantly better seizure outcomes than ASMs and KDTs. The most effective method in providing seizure-free status was hemispherectomy. Moreover, multimodal treatments provided significant improvements in EEG status and neurodevelopment. Improvements in severe EEG abnormalities have been observed both among seizure-free patients and in patients with persistent seizures [21].

In a long-lasting retrospective study on the use of ASMs, KDTs, and surgical methods, Kim et al. [22] reported similar findings. The authors studied the medical records of 68 patients with LGS, all initially treated with ASMs. In this cohort, 26 patients received ASMs alone, and 42 patients were treated with additional therapies, including 11 patients treated with two additional therapies, and 6 patients treated with three additional therapies. A total of 19 patients were prescribed KDTs, 15 underwent focal resective surgery, 17 underwent CC, and 14 underwent VNS. Of the 68 patients in the final follow up phase, 23.5% were seizure-free for a mean follow-up time of 19.3 years. Of the aforementioned methods, focal resective surgery yielded the best results, as more than half of the patients achieved seizure-free status. Similar to previous authors, Kim et al. [22] reported improvements in EEG, as DSSW and GFPA disappeared in more than half of the patients following a multimodal course of treatment.

Another study on several treatment methods for LGS was conducted by Reyhani et al. [42]. These authors studied the population of 20 adult patients treated with various methods, including VNS (*n* = 10), KDTs (*n* = 2), CC (*n* = 1), and resective surgery with subsequent CC (*n* = 1). The results observed in this cohort are generally unsatisfactory, with none of the patients achieving seizure freedom [42]. The results of this study are consistent with the widespread view that adult patients with LGS have poorer treatment outcomes because they are less responsive to treatment [26], are often misdiagnosed [97], and may experience more adverse effects during CC [28].

A recent review by Samanta et al. [16] evaluated the effectiveness of LGS treatment options for distinct seizure types. They found that atonic seizures respond particularly to CC and VNS, and CC outperforms VNS. On the other hand, GTCS, atypical absence, and myoclonic seizures respond well to pharmacological treatment, and myoclonic seizures respond poorly to CC and neuromodulation methods [16].

The following are studies comparing each pair of treatment methods.

### 5.1. Corpus Callosotomy and Vagus Nerve Stimulation

For collective seizure reduction, a recent meta-analysis by Ferreira Soares [98] comparing CC and VNS showed that both methods are beneficial in the palliative treatment of LGS, as seizure reduction is achieved in most patients. No statistically significant advantage was reported between CC and VNS in terms of seizure reduction rates of both ≥50% and >0% [98]. In terms of specific seizure types, CC has been found to be particularly effective in atonic seizures [41], while it has been suggested that VNS is more effective when GTCS, atypical absence, and myoclonic seizures predominate [41]. In a meta-analysis regarding distinct seizure types, Lancman et al. [99] revealed that CC was significantly more effective in providing at least 50% and 75% reduction in atonic seizures, with no further statistically significant differences.

It was estimated that compared to CC, VNS is more cost-effective in annual outcomes for patients with LGS. This method continues to be more cost-effective for about 20 years after surgery for atonic seizures and even more so for other types of seizures, which are more difficult to treat with CC. It is therefore the case that VNS is worth considering as first-line therapy [100]. Moreover, the therapeutic effects of VNS tend to progress over time, unlike those of CC [97,100].

### 5.2. Corpus Callosotomy and Ketogenic Dietary Therapies

A 2021 meta-analysis by Sharawat et al. [6] compared CC to KDTs based on studies involving 436 and 185 patients with LGS. CC was shown to be more effective in achieving seizure freedom, at least 75% seizure reduction, and 50% seizure reduction, respectively [6]. CC is also more effective in controlling drop seizures [16]. On the other hand, CC was associated with more short-term adverse effects [6].

### 5.3. Open Corpus Callosotomy and Stereotactic Laser Ablation Corpus Callosotomy

A recent meta-analysis on the DRE patient population suggests that traditional open CC and laser interstitial thermal therapy (LITT) provide similar efficacy. The authors found comparable rates of seizure-free patients (19.95% for open CC and 14.5% for LITT, respectively), of drop attack freedom (45.1% and 51.3%, respectively), and >50% seizure reduction (55.2% and 52.6%, respectively). Complication rates were also comparable, but LITT was linked to shorter hospitalization. Of note, the diagnosis of LGS was a predictor of seizure freedom [101]. 

### 5.4. Vagus Nerve Stimulation, Deep Brain Stimulation, and Responsive Neurostimulation

In a recent meta-analysis comparing the efficacy of VNS, DBS, and RNS in LGS patients, the pooled responder rate reached 55.4%. The greatest responder rate was provided by DBS (69.7%), followed by RNS (63.0%), and VNS (50.6%) [72]. 

On the other hand, DBS was linked to the highest rate of severe adverse effects, which may limit its use early in the treatment algorithm [72]. 

RNS was suggested as a good therapeutic option for patients with suboptimal response to VNS [80]. VNS was linked to the greatest tendency towards stimulation-related adverse effects, however, they are often tolerable [72].

### 5.5. Data from RCTs on Anterior Corpus Callosotomy, Deep Brain Stimulation, and Antiseizure Medications in Drop Seizure Control

In 2025, Zhu et al. [74] published a meta-analysis of RCTs on pharmacological and non-pharmacological LGS therapies. They assessed the efficacy based on the reduction in drop seizures (at least 50% and median). They found that the three most efficacious modalities providing at least a 50% reduction in drop seizures were clobazam, anterior CC, and rufinamide. The same treatments also showed a beneficial safety profile, with high-dose clobazam (1 mg/kg/day) being safe and the most effective treatment. The greatest drop seizure reduction was seen in patients using clobazam, rufinamide, and DBS (parameter not available in RTC on CC). Among the compared treatments, the lowest risk of adverse effects and serious adverse effects was presented by DBS. However, the authors stated that due to small study populations, the data on CC and DBS require further confirmation [74]. 

### 5.6. The Proposed LGS Treatment Algorithms

As treatment of LGS is one of the most challenging among DREs, the development of a standardized treatment protocol posed a major challenge [21]. However, several authors provided attempts to create a reference treatment algorithm. 

In 2017, Cross et al. [53] provided an expert opinion on LGS treatment based on the literature search and their experience, and later updated their algorithm in 2024 [102]. According to their protocol, for newly diagnosed LGS, the proposed first-line therapy was valproate [53,102]. As valproate is highly teratogenic, risk–benefit ratio should be carefully balanced in each female patient with childbearing potential [102]. Then, lamotrigine should be added in case of valproate ineffectiveness (in reduced dose, as valproate inhibits its metabolism) [53,102]. Third-line ASM treatment relied on the addition of rufinamide, with attempts to discontinue valproate (if so, increase lamotrigine dose) or lamotrigine [53,102]. In case of further pharmacoresistance, alternative ASMs should be used after consultation with the patient/caregivers (particularly topiramate [53,102], clobazam [53,102], felbamate [53,102], cannabidiol [102], or fenfluramine [102]). 

Along with pharmacotherapy, non-pharmacological therapy should be considered [53]. KDTs can be considered early, while the use of VNS and CC relies largely on the decision of patients/caregivers. Resective and disconnection surgery “must be considered in all patients, particularly those with LGS with structural etiology” [102]. Early CC should be particularly recommended for patients suffering mainly from drop attacks [53,102]. 

Apart from the treatment algorithm, they recommended regular reassessments of treatment plans, minimization of polytherapy, active search for adverse effects in patients, careful management of comorbidities, and the use of protective equipment if indicated. As the parallel use of three or more ASMs provides no documented benefits while enhancing the risk of adverse effects, a goal of no more than two ASMs at a time should be maintained [53,102]. 

In 2025, Warren et al. [43] summarized content on LGS treatment presented at the Pediatric State of the Art Symposium 2024 (American Epilepsy Society annual meeting). The work added several useful comments, underlining the promising role of DBS, the problem of often delayed surgery, lack of head-to-head ASMs studies, and the low/very low quality of studies on ASMs other than valproate, lamotrigine, and rufinamide [43].

## 6. Conclusions

Since many patients with LGS develop pharmacoresistance, several non-pharmacological therapies have been investigated for the treatment of the disease.

On the one hand, surgical therapies are still the mainstay of therapy in patients with LGS refractory to pharmacotherapy because they provide significant seizure reduction. Among candidates for surgical treatment, patients with an epileptogenic zone detectable via non-invasive diagnostic methods (PET, SPECT, and MEG) seem to be in the most favorable situation. When non-invasive methods fail to detect the epileptogenic zone, invasive methods are considered, including SEEG and icEEG. SEEG is reported to have a lower incidence of complications compared to icEEG. In icEEG, the use of depth electrodes appears to be associated with fewer complications compared to subdural electrodes. If SEEG is considered, 5-SENSE can be used to predict failure to detect a focal seizure-onset zone. 

If the primary epileptogenic areas can be localized, resective surgery is the first-line treatment, although in many patients with LGS, the primary epileptogenic zone cannot be detected. CC remains the most popular method of palliative surgery. Although total CC is associated with better seizure reduction, partial CC is preferred as first-line CC due to its lower complication rate. Minimally invasive CC methods, including SLACC, and SRS CC, appear to be safer and similarly effective compared to traditional open CC. 

VNS is a viable surgical option for patients with LGS due to its effectiveness and tolerability. Efficacy is demonstrated by reduced seizure frequency and ictal or postictal severity, as well as improved QOL, which might be independent of the reduction in the number of seizures. However, seizure reduction results are inconsistent across seizure types, and it would be beneficial to select patients based on the impact of seizure type on QOL and expected response to treatment. Although improvements in higher-order functions and QOL might vary among individuals, VNS treatment cannot impair them. In addition, mental retardation may be a negative prognostic factor for VNS treatment. A “honeymoon effect,” although rare and not clearly understood, may be anticipated and may improve patients’ and caregivers’ initial approach to treatment. Another potential benefit of VNS may be a reduction in the number of ASMs, and therefore, fewer side effects of polytherapy, which, however, usually must be maintained despite VNS treatment. In addition to the duty cycle, other appropriate stimulation parameters should be established for LGS patients, not only to improve efficacy, but also to avoid adverse effects such as the observed worsening of seizure frequency as a result of the increase in current intensity. Compared with CC, VNS therapy has a higher tolerability, and when used in combination with CC, it is more effective when performed first, although not for all seizure types. Due to their greater effectiveness, auto-stimulation VNS models and RDC are worth considering, although data regarding these methods are limited.

In addition to the above-mentioned surgical treatment, KDTs are worth considering as they can provide significant seizure reduction, including seizure-free status, with minimal risk of adverse effects. The most commonly used dietary therapy is a cKD, although due to its comparative restrictiveness and requirements for trained dieticians, alternative diets such as MAD and LGIT may be considered.

Furthermore, because combinations of individual treatment options can provide dramatic reduction in seizure number, the literature review highlights the significant benefits of multimodal LGS treatment. When choosing a particular method, physicians should also rely on recommended treatment algorithms. Patients should be regularly reevaluated depending on the actual treatment option.

Since seizure diaries reported by caregivers are time-consuming and presumably inaccurate (due to difficulties in the recognition of seizures, especially in LGS patients), the use of objective, fast, and reliable biomarkers such as GPFA may be recommended for future trials. Additionally, such biomarkers may facilitate the optimization of treatment by allowing rapid titration of treatment parameters. 

Given the diversity of approaches to data collection, definitions of responder groups, classifications of seizure reduction rates and QOL, and duration of follow-up, there may be a need for standardization to facilitate comparison of these factors in future studies.

## Figures and Tables

**Table 1:** The effectiveness of resective surgery and additional surgical therapies regarding Engel scale-measured seizure reduction.

Surgical Method	Age at Surgery	LGS Patients, *n*	Time of Assessment/Duration of Follow-Up	Engel Class, %				Comments	Reference, Study Type
				IV	III	II	I		
Resective surgery overall	9.3 ± 4.4 years, mean ± SD	90	6.1 ± 2.2 years	33.3	7.8	8.9	50.0	Adverse effect: minor bleeding	Kang et al. [17], (R)
Hemispherectomy	N/A	21	N/A	14.3	9.5	4.8	71.4		
Single lobe resection	N/A	18	N/A	44.4	5.6	11.1	38.9		
Multilobar resection	N/A	51	N/A	37.3	7.8	9.8	45.1		
Surgery overall	11.5 years	18	mean of 5.4, range 1–9 years	11.1	22.2	27.8	38.9	Adverse effects: fever (less than one week), acute disconnection syndrome, partial aphasia, contralateral partial hemiplegia (each less than 3 weeks), and contralateral hemianopia.	Liu et al. [18], (R)
Single lobe resection	N/A	2	8 years *, mean (7 and 9 years)	0.0 *	0.0 *	0.0 *	100.0 *		
Lesionectomy + MST	N/A	1	7 years	0.0 *	100.0 *	0.0 *	0.0 *		
Multilobar resection + MST	N/A	11	4.36 years *, mean (range 1–8)	9.1 *	18.2 *	27.3 *	45.5 *		
Multilobar resection + MST + CC	N/A	4	6.75 years *, mean (range 5–9)	25.0 *	25.0 *	50.0 *	0.0 *		
Resective surgery overall	7.8 ± 3.7 years, mean ± SD	27	33.1 ± 20.3 months	18.5	7.4	14.8	59.3	Adverse effects: hemianopia, hemiplegia, minor infarction around the motor cortex with subsequent weakness of the contralateral upper extremity (all recovered within 6 months), and postoperative with subsequent hematomas (absorbed spontaneously).	Lee et al. [19], (R)
Single lobe resection	7.7 years, mean	11	N/A	36.4	9.1	9.1	45.5		
Multilobar resection	8.3 years, mean	10	N/A	10.0	10.0	20.0	60.0		
Hemispherectomy	7.5 years, mean	6	N/A	0.0	0.0	16.7	83.3		
Exclusively resective surgery	9.70 ± 3.66 years	20	1st year	15.0 *	5.0 *	15.0	65.0	First prospective study concerning the effects of resective surgery combined with CC among LGS pediatric patients without focal lesions showed on brain MRI. Adverse effects: urinary incontinence, hemiplegia, aphasia, and apraxia (among the whole study population). All subsided within 3 weeks.	Ding et al. [20], (P)
		20	3rd year	N/A **	N/A **	N/A **	55.0		
		16	5th year	10.0 *	20.0 *	10.0 *	40.0 *		
Resective surgery + anterior CC	9.48 ± 3.88 years	23	1st year	4.3 *	8.7 *	13.0	73.9		
		23	3rd year	8.7 *	8.7 *	17.4	65.2		
		17	5th year	13.0 *	4.3 *	13.0 *	43.5 *		
Single lobe resection	N/A	27	1st year	N/A	N/A	N/A	40.7 ***	-	Na et al. [21], (R)
			5th year	N/A	N/A	N/A	11.1 ***		
Multilobar resection	N/A	60	1st year	N/A	N/A	N/A	53.3 ***		
			5th year	N/A	N/A	N/A	16.7 ***		
Hemispherectomy	N/A	25	1st year	N/A	N/A	N/A	52.0 ***		
			5th year	N/A	N/A	N/A	24.0 ***		
Resective surgery overall	17.3 ± 4.4 years, mean	15	5.3 ± 2.3 years	N/A	N/A	46.7 *	20.0 *	One patient (Engel II) achieved Engel I class after another resective surgery (included as Engel II).	Kim et al. [22], (R)

*—value calculated based on publication data; **—inconsistent data; ***—in the original publication, the value was presented as seizure-free status (which corresponds with Engel I). Abbreviations: CC—corpus callosotomy; LGS—Lennox–Gastaut syndrome; MRI—magnetic resonance imaging; MST—multiple subpial transection; N/A—not available; (P)—prospective; (R)—retrospective.

**Table 2:** The effectiveness of corpus callosotomy regarding percentage seizure reduction.

Surgical Method	Age at Surgery	LGS Patients, *n*	Time of Assessment/Duration of Follow-Up	Responders, %	Seizure-Free Status	Comments	Reference, Study Type
CC and subsequent VNS	1–28, and 3–30 years, respectively	10	12th month after VNS	60.0 ^a^	20.0	Adverse effects: CC—acute disconnection syndrome (stranguria and mutism that resolved 3–7 days after surgery); VNS—transient hoarseness, coughing.	Katagiri et al. [8], (R)
CC	N/A	100	1st year	N/A	24.0	-	Na et al. [21], (R)
			5th years	N/A	11.0		
CC	16.3 ± 5.3 years, mean	17	At the last available follow-up (mean of 6.1 ± 3.9 years)	52.9 *^a^	5.9 *	Five patients achieving ≥90% seizure reduction were free of GTCS and drop attacks.	Kim et al. [22], (R)
SLACC	33 years, median	10	mean of 19 months, range 6–40 months	80.0 ^a^	20.0	Adverse effects: asymptomatic intracerebral hemorrhage, hypersomnia, and aggressiveness development.	Tao et al. [24], (R)
Anterior CC	9.48 ± 2.21 years	23	1st year	87.0 ^a^	17.4	Adverse effects: urinary incontinence, aphasia, apraxia (all transient).	Liang et al. [25], (P)
		23	2nd year	69.6 *^a^	13.0		
		23	5th year	65.2 *^a^	8.7		
SLACC	35.2 years *, mean	6	1st year	83.3 *^d^	33.3 *	Severe intraparenchymal hemorrhage resulting in the procedure’s cessation in one case (included in the table). Lacking data regarding one of the seizure types could lower the overall response rate (2 patients). Adverse effects: severe intraparenchymal hemorrhage; transient: leg weakness, hemiparesis, incontinence, dysarthria, SMA syndrome; persistent: dysarthria, hemiparesis, and incontinence.	Rich et al. [28], (R)
	33.3 years *, mean	7	N/A ***	71.4 *^d^	14.3 *	-	
SLPCC	23.7 years *, mean	3	1st year	33.3 *^d^	33.3 *	Lacking data regarding one of the seizure types could lower the overall response rate (2 patients). Adverse effect: persistent agraphia.	
		3	N/A ***	66.7 *^d^	0.0 *	-	
Total CC	10.5 ± 5.3 years, mean ± SD	18	mean of 29.2 ± 12.4 months, range 12–54 months	N/A	10.0	After CC, the patients achieved “average 60–70% reduction in all seizure types” with a complete cessation of drop attacks. Adverse effects: disconnection syndrome-like symptoms (“confusion, increased response time latency and apathy”; the symptoms “improved to pre-surgical level by the time of discharge”.	Kurwale et al. [31], (R)
Anterior half CC	11.2 ± 3.02 years **	15 + 9 LGS-like	2nd year	N/A for all seizure types collectively	10.0	Responders regarding a specific seizure type: 90.0% in atonic, 20.0% in tonic, 10.0% in myoclonic, 93.0% in atypical absence, and 40.0% in GTCS. Adverse effects: acute disconnection syndrome (apathy, urinary incontinence, non-dominant hemineglect lasting up to 3 weeks).	Cukiert et al. [32], (P)
SRS CC (genu, first third of the truncus)	22 years	1	18th month	100.0 *^a^	0.0 *	First literature reports of SRS CC. Adverse effect: transient headache.	Pendl et al. [37], (R)
SRS CC (anterior)	28 years	1	18th month	100.0 *^a^	0.0 *		
SRS CC (1/3 anterior)	14 years	1	N/A	0.0 *^a^	0.0 *	The patient experienced aggressive behavior alleviation, while seizure status became unaffected. No adverse effects reported	Eder et al. [38], (R)
SRS CC (posterior, completion)	20 years	1	8th month	N/A	N/A	Open 2/3 ACC prior to SRS CC procedure. The patient achieved marked improvement in seizure frequency and quality, he became ambulatory and free of atonic seizures. No adverse effects reported.	Sachdev et al. [39], (R)
Transventricular total CC	5 years	1	1st month	100% *^a^	100% *	Adverse effect: pseudomeningocele.	Baumgartner et al. [40], (R)
		1	2.5th year	N/A	N/A		
Surgical CC	9.6 ± 1.1 years, mean	5	mean of 15.2 ± 3.6 months	60.0 ^c^	0.0	Adverse effect: cerebrospinal fluid leakage.	Alanazi et al. [41], (R)
Focal resective surgery followed by CC	N/A	1	N/A	0.0 *^a^	0.0 *	The study comprised 20 adult LGS patients, of whom two were administered surgical procedures. No seizure benefits were observed among the two surgical patients.	Reyhani et al. [42], (R)
CC	N/A	1	N/A	0.0 *^a^	0.0 *	-	

Responders defined as patients achieving: ^a^—≥50% seizure reduction/ not specified by the authors; ^c^—>50% seizure reduction overall; ^d^—>50% seizure reduction in astatic seizures or GTCS (“meaningful improvement”). *—value calculated based on publication data; **—inconsistent data in the publication; ***—at last available follow-up. Abbreviations: CC—corpus callosotomy; GTCS—generalized tonic-clonic seizures; LGS—Lennox–Gastaut syndrome; N/A—not available; (P)—prospective; (R)—retrospective; SLACC—stereotactic laser anterior corpus callosotomy; SLPCC—stereotactic laser posterior corpus callosotomy; SRS—stereotactic radiosurgery; and VNS—vagus nerve stimulation.

**Table 3:** The effectiveness of neurostimulation therapies regarding percentage seizure reduction.

Age at Surgery	LGS Patients, *n*	Time of Assessment/Duration of Follow-Up	Responders, %	Seizure-Free Status, %	Comments	Reference, Study Type
VNS						
N/A	35	1st year	N/A	14.3	-	Na et al. [21], (R)
		5th year	N/A	2.9		
18.1 ± 4.9 years, mean	14	mean 5.4 ± 2.2 years	N/A	7.1 *	-	Kim et al. [22], (R)
8.6 ± 3.2 years ***, mean	12 + 8 LGS-like	2nd year	N/A for all seizure types collectively	0.0	Responders regarding a specific seizure type ^e^: 20.0% in atonic, 40.0% in tonic, 63.6% in myoclonic, 80.0% in atypical absence, and 46.2% in GTCS. Adverse effects: hoarseness in one patient.	Cukiert et al. [32], (P)
11.7 ± 3.3 years, mean	4	24.5 ± 16.5 months	50.0 *^a^	0.0	Adverse effect: swallowing difficulty in one patient.	Alanazi et al. [41], (R)
N/A	10	N/A	50.0 *^a^	0.0	The study comprised 20 adult LGS patients, of whom 10 were administered VNS	Reyhani et al. [42], (R)
23.0 years (IQR 14.5–29.5), median	29	median 66 months (IQR 42.5–126.5)	41.4 *^a^	0.0	VNS was turned off in 7 patients due to the lack of efficacy. Adverse effects: cough, sore throat, and hoarseness (moderate and reversible).	Suller Marti et al. [54], (R)
8.4 years (5.0–12.0), mean	14 + 10 LGS-like	mean 32 months (24–53)	83.3 *^b^	N/A	“Honeymoon effect” in 58.3% of the patients; one withdrawal due to parkinsonian symptoms; one case of persistent hoarseness. Adverse effects: seizure frequency worsening (transient).	Cukiert et al. [56], (P)
20.8 months, mean	26	≤1st month	38.5 *^c^	11.5 *	A total of 71 patients enrolled in the study. Adverse effects: dysphonia, paresthesia, and shortness of breath (well tolerated, resolved after 24 months).	Abdelmoity et al. [58], (R)
	32	3rd month	43.8 *^c^	9.4 *		
	44	6th month	54.5 *^c^	11.4 *		
	37	12th month	67.6 *^c^	10.8 *		
	24	18th month	66.7 *^c^	20.8 *		
	23	24th month	65.2 *^c^	17.4 *		
13.0 years (4.0–52.0), median	30	median 52 months (17–123 months)	66.7 *^a^	3.3 *	The adverse effects occurred in 20 patients, typically after the increase in the output current. Five patients (16.7%) withdrew from VNS therapy either due to adverse events or lack of clinical benefit. Adverse effects: drooling, voice alteration; one case of vocal cord paralysis (disappeared with device’s switch-off and forced the device explanation).	Kostov et al. [59], (R)
13.0 years (5.0–27.0), median	46	1st month	43.5 *^a^	0.0	Declining number of patients was due to data-collection cutoff point. Adverse effects: voice alteration, hoarseness, and coughing (resolved after stimulation adjustment).	Frost et al. [60], (R)
	43	3rd month	55.8 *^a^	N/A		
	24	6th month	58.3 *^a^	N/A		
11.2 years (6.3–18.8), mean	17	24th month	23.5 *^c^	N/A	One patient excluded due to VNS equipment failure and one patient withdrew consent (thus 17 patients were eventually analyzed).	Aldenkamp et al. [61], (P)
13.0 years (5.0–19.5), mean	46	mean 30 months (12–108)	65.2 */87.0 *^a^***	0.0	Adverse effects: hoarseness and coughing, change in vocal timbre.	Cersósimo et al. [62], (R)
N/A	123	6th month	28.5 *^d^	N/A	Adverse effects: device-related (lead’s damage, device change, device removal, infection, and lead issues) and dysphonia.	Orosz et al. [63], (R)
	146	12th month	32.9 *^d^			
	87	24th month	39.1 *^d^			
DBS						
26 years, mean	3	mean 3.8 years	100 *^a^	33.3 *	ANT DBS was used in all three patients.	Poulen et al. [67] (R)
24.4 ± 6.92 years	10	3rd month	50.0 ^a^/89.0 ^f^	0.0	RCT.. Stimulation target—CMT. Adverse effects: transient drowsiness in 60% of the patients (postoperative), transient ipsilateral hand/face/lip paresthesia (stimulation-related).	Dalic et al. [69] (P)
13.2 years, mean	13	18th month	92.3 ^a^	15.3	CMT DBS was used in all 13 patients. Adverse effects: two cases of skin erosions and one case of rupture of electrode lead and connector cable—all requiring electrode explanation.	Velasco et al. [71] (R)
14 years, mean	6	16.3 months, mean	66.7 *^g^	16.7 *	CMT DBS was used in all six patients.	Bonda et al. [73] (R)
RNS						
16 years	1	26 months	100 *^a^	0 *	18 months after RNS placement, the right fronto-polar lead was replaced by the right CMT depth electrode.	Kwon et al. [76], (R)
12 years	1	1 year	100 *^a^	0 *	After RNS placement, the patient was able to walk independently.	
12.2 years	10	At the last available follow-up (N/A)	60 ^h^	10 *	-	Ahn et al. [78] (R)
15.5 years	4	At the last available follow-up (24.8 months, mean)	75 *^a^	0 *	-	Roa et al. [79], (R)
16.3 years (RNS), 11.5 years (VNS)	4	3.1 years (RNS, mean), 7.8 years (VNS, mean)	100 *^a^	0 *	All patients were treated with RNS and VNS concomitantly.	Beaudreault et al. [80] (R)
17 years	1	N/A	100 *^a^	0 *	-	Zuckerman et al. [83] (R)

Responders defined as patients achieving: ^a^—≥50% overall seizure reduction; ^b^—≥50% seizure reduction in any specific seizure type; ^c^—>50% overall seizure reduction; ^d^—≥50% reduction in the predominant (the most disabling) seizure type; ^e^—≥50% seizure reduction for each seizure type separately; ^f^—≥50% seizure reduction in electrographic seizures; ^g^—>66% seizure reduction; ^h^—≥50% seizure reduction in the most disabling seizure type. *—value calculated based on publication data; ***—inconsistent data in the publication. Abbreviations: ANT—anterior nucleus of the thalamus; CMT—centromedian nucleus of the thalamus; DBS—deep brain stimulation; GTCS—generalized tonic-clonic seizures; IQR—interquartile range; LGS—Lennox–Gastaut syndrome; N/A—not available; (P)—prospective; (R)—retrospective; RCT—randomized controlled trial; RNS—responsive neurostimulation; and VNS—vagus nerve stimulation.

**Table 4:** Diet components and requirement for hospitalization in example ketogenic diet therapies.

Parameter	Classic Ketogenic Diet 4:1#	Medium Chain Triglyceride Diet 1.9:1#	Modified Atkins Diet 0.8:1#	Low Glycemic Index Treatment 2:3#
% of daily calories intake from fat	90% [88,92]	50% from MCT + 21% from LCT [92]	65% [92]	60% [92]
% of daily calories intake from carbohydrates	4% [92]	10% [92]	3–6% [92]	12% (2), with a preference of GI < 50 [88]
% of daily calories intake from protein	6% [92]	19% [92]	29–32% [92]	28% [92]
Requirement for hospitalization	Usually yes [88]	Usually yes [88]	No [88]	No [88]

Abbreviations: GI—glycemic index; LCT—long chain triglycerides; MCT—medium chain triglycerides; #—ketogenic ratio; and the exemplary ketogenic ratio of 4:1 sets a proportion of 4 g of fat per 1 g of carbohydrate + protein.

**Table 5:** The effectiveness of ketogenic dietary therapies regarding percentage seizure reduction.

KDT Protocol	Age at Initiation of Diet Therapy	LGS Patients, *n*	Time of Assessment/Duration of Follow-Up	Responders, %	Seizure-Free Status, %	Comments	Reference, Study Type
cKD = 18 (4:1# = 16, 3:1# = 2); MAD = 2	4.6 years, median	20	3rd month	25.0 *^a^	0.0 *	The study concerned LGS connected to mitochondrial disease. Four patients initially treated with cKD 4:1 switched to cKD 3:1 during the study. Adverse effects: poor oral intake, vomiting, diarrhea, and metabolic acidosis (no life-threatening events).	Na et al. [5], (R)
		13	6th month	45.0 *^a^	5.0 *		
		9	9th month	35 *^a^	10.0 *		
		9	12th month	35 *^a^	10.0 *		
		8	24th month	35 *^a^	10.0 *		
KDT, not specified	11.5 ± 4.9 years, mean	19	N/A	31.6 *^d^	26.3 *	-	Kim et al. [22], (R)
		6	After KDT discontinuation, N/A	26.3 *^d^	0.0 *		
		6	N/A **	26.3 *^d^	5.3 *	One of the patients regained seizure free-status after lobectomy (included in ≥50% seizure reduction status, but not in seizure-free status), another one regained seizure-free status due to continued MAD administration (included in ≥50% seizure reduction status and in seizure-free status).	
201 (cKD = 169, MAD = 27, LGIT = 5)	N/A	201	1st year	N/A	26.4	-	Na et al. [21], (R)
		N/A	5th year	N/A	7.5		
KDT, not specified	N/A	2	At least 1 year, N/A **	0.0 ^a^	0.0	The study comprised 20 adult LGS patients, of whom 2 were administered KDTs. One of the patients achieved a 40% seizure reduction, with no seizure reduction in the second patient.	Reyhani et al. [42], (R)
MAD	N/A	7	4th week	28.6 ^b^	14.3	Adverse effects in the whole studied population (n = 52): digestive symptoms, asymptomatic hypoglycemia, significant sleep increase, and abnormal liver function.	Wu et al. [86], (P)
		7	12th week	42.9 ^b^	14.3		
		2	24th week	14.3 *^b^	14.3 *		
cKD 4:1#	4.4 ± 3.2, mean ± SD	47	1st month	36.2 *^a^	4.3	Adverse effects: hyperlipidemia, gastrointestinal, fatigue, and drowsiness.	Zhang et al. [93], (R)
		47	3rd month	49.0 *^a^	4.3		
		40	6th month	44.7 *^a^	8.5 *		
MAD	4.5 years, median	25	3rd month	48.0 ^c^	8.0	Adverse effects: constipation, vomiting, and anorexia.	Sharma et al. [95], (R)
		11	6th month	44.0 ^c^	12.0		
		9	12th month	36.0 ^c^	12.0		
LGIT	N/A	12	12th month	75.0 ^a^	0.0	Adverse effects in the whole study population (n = 36): hypercholesterolemia, reduced tCO2, increased ALT, increased lipase and amylase, increased BUN, and increased blood creatinine.	Kim et al. [96], (R)

Responders defined as patients achieving: ^a^—≥50% seizure reduction/not specified by the authors; ^b^—Engel I, II, III, and IV grades (here describes as <50%, 50–90%, 90–100% seizure reduction, and seizure-free status, respectively); ^c^—>50% seizure reduction overall; ^d^—≥50% seizure reduction in the predominant seizure type. *—value calculated based on publication data; **—at last available follow-up. #—Ketogenic ratio (fat:carbohydrate + protein mass ratio). Abbreviations: ALT—alanine aminotransferase; BUN—blood urea nitrogen; cKD—classic ketogenic diet; DFD—drug-free diet; KDT—ketogenic dietary therapy; LGIT—low glycemic index treatment; LGS—Lennox–Gastaut syndrome; MAD—modified Atkins diet; N/A—not available; (P)—prospective; (R)—retrospective; and tCO2—total carbon dioxide.

## Data Availability

Not applicable.

## Abbreviations

5-SENSE	5-SENSE score calculator
AAN	American Academy of Neurology
ALT	Alanine aminotransferase
ANT	Anterior nucleus of the thalamus
ASMs	Antiseizure medications
BUN	Blood urea nitrogen
CC	Corpus callosotomy
cKD	Classic ketogenic diet
CMT	Centromedian nucleus of the thalamus
CNS	Central nervous system
CPS	Complex partial seizures
DBS	Deep brain stimulation
dDTF	Direct directed transfer function
DFD	Drug-free diet
DQ	Developmental quotient
DRE	Drug-resistant epilepsy
EEG	Electroencephalography
ESTEL	Electrical stimulation of thalamus for epilepsy of Lennox–Gastaut phenotype
FDA	Food and Drug Administration
FIRES	Febrile infection related epilepsy syndrome
GGE	Genetic generalized epilepsy
GI	Glycemic index
GLUT-1	Glucose transporter 1
GPFA	Generalized paroxysmal fast activity
GSSW	Generalized slow spike-and-wave
GTCS	Generalized tonic-clonic seizures
HIE	Hypoxic ischemic encephalopathy
icEEG	Intracranial electroencephalography monitoring
IQ	Intelligence quotient
IQR	Interquartile range
KDT	Ketogenic dietary therapy
LCT	Long chain triglycerides
LGIT	Low glycemic index treatment
LGS	Lennox–Gastaut syndrome
LITT	Laser interstitial thermal therapy
MAD	Modified Atkins diet
MCT	Medium chain triglyceride (or medium chain triglyceride diet)
MEG	Magnetoencephalography
MQ	Memory quotient
MRI	Magnetic resonance imaging
MSNPE	Myoclonic status in non-progressive encephalopathy
MST	Multiple subpial transection
MVAR	Multivariate autoregressive modeling
NHBI	Neonatal hypoglycemic brain injury
PET	Positron emission tomography
QOL	Quality of life
RCT	Randomized controlled trial
RDC	Rapid duty cycle
RNS	Responsive neurostimulation
SEEG	Stereoencephalography
SLACC	Stereotactic laser anterior corpus callosotomy
SLCC	Stereotactic laser corpus callosotomy
SLPCC	Stereotactic laser posterior corpus callosotomy
SPECT	Single-photon emission computed tomography
SQ	Social quotient
SRS	Stereotactic radiosurgery
tCO2	Total carbon dioxide
taVNS	Transauricular vagus nerve stimulation
tcVNS	Transcervical vagus nerve stimulation
VNS	Vagus nerve stimulation
